# The Synergistic Effects of *Corbicula fluminea* and *Sarcodia montagneana* on Alleviating Systemic Inflammation and Osteoarthritis Progression

**DOI:** 10.3390/antiox12122068

**Published:** 2023-12-01

**Authors:** Tse-Hung Huang, Bang-Hung Liu, Chia-Hui Hsu, Chang-Jer Wu, Kuang-Wen Liao, Chen-Si Lin, Yi-Lin Chan

**Affiliations:** 1 Department of Traditional Chinese Medicine, Linkou Chang Gung Memorial Hospital, Taoyuan 33305, Taiwan; kchuang@cgmh.org.tw; 2 School of Traditional Chinese Medicine, Chang Gung University, Taoyuan 33303, Taiwan; 3 Research Center for Food and Cosmetic Safety, Chang Gung University of Science and Technology, Taoyuan 33303, Taiwan; 4 Research Center for Chinese Herbal Medicine, Chang Gung University of Science and Technology, Taoyuan 33303, Taiwan; 5 Department of Chemical Engineering and Graduate Institute of Biochemical Engineering, Ming Chi University of Technology, New Taipei City 24301, Taiwan; 6 Department of Veterinary Medicine, National Taiwan University, Taipei 10617, Taiwan; 2309258@narlabs.org.tw; 7 Center for Animal Health and Food Safety, College of Veterinary Medicine, University of Minnesota, Saint Paul, MN 55108, USA; hsu00124@umn.edu; 8 Department of Food Science and Center of Excellence for the Oceans, National Taiwan Ocean University, Keelung 20224, Taiwan; cjuw@mail.ntou.edu.tw; 9 Department of Biological Science and Technology, National Yang Ming Chiao Tung University, Hsinchu 30068, Taiwan; liaonms@nycu.edu.tw; 10 Department of Life Science, Chinese Culture University, Taipei 11114, Taiwan

**Keywords:** osteoarthritis, *Sarcodia montagneana*, *Corbicula fluminea*, MIA-induced mouse model, tumor necrosis factor-α

## Abstract

Osteoarthritis (OA) is a progressive disease that causes pain, stiffness, and inflammation in the affected joints. Currently, there are no effective treatments for preventing the worst outcomes, such as synovitis or cartilage degradation. *Sarcodia montagneana* and *Corbicula fluminea* are common species found in the ocean or in freshwater areas. Their extracts are demonstrated to possess both antioxidative and anti-inflammatory functions. This study aimed to investigate the synergistic effects of the extracts of *Sarcodia montagneana* (SME) and *Corbicula fluminea* (FCE) on reducing local and systemic inflammation, as well as their efficacy in OA symptom relief. An in vitro monocytic LPS-treated THP-1 cell model and in vivo MIA-induced mouse OA model were applied, and the results showed that the combinatory usage of SME and FCE effectively suppressed IFN-*γ* and TNF-α production when THP-1 cells were treated with LPS. SME and FCE also significantly decreased the systemic TNF-α level and joint swelling and prevented the loss of proteoglycan in the cartilage within the joints of OA mice. The data shown here provide a potential solution for the treatment of osteoarthritis.

## 1. Introduction

Degenerative osteoarthritis (OA) is a progressive disorder that affects millions of people worldwide, especially those aged 60 years or older [1]. The disease affects the articular cartilage of the synovial joints and is characterized by joint inflammation and changes in the tissue architecture, including the destruction of cartilage, subchondral bone remodeling, and the formation of osteophytes. Although OA has a multifactorial etiology, including genetic and environmental components, risk factors such as obesity, joint injury, and aging can cause irreversible damage to the joints [2]. Treatment of OA involves disease-modifying drugs, symptom control, and surgical interventions, such as joint replacement [3]. However, these treatments have limitations, and thus, major efforts have focused on the identification of novel therapeutic targets.

LPS and mono-iodoacetate (MIA)-induced osteoarthritis (OA) models are reliable animal models for evaluating the degree of laboratory animal osteoarthritis and the level of the inflammatory system within the knee joint [4]. Compared to surgical interventions on the knee joint, the MIA-induced model is easier to use and has consistent outcomes. In acute arthritis research, a standardized scoring system, namely, the Osteoarthritis Research Society International (OARSI) score, has been widely adopted for assessing OA in rats and mice at the macroscopic and microscopic levels [5]. Through the systemic classification of different grades and stages of histopathology assessment, OARSI is a useful pathological guideline. It is able to quantitatively describe the degree of degeneration in the knee joint and inflammatory status. This scoring system could also be a proxy indicator of the damage or degeneration property between cartilage and bone tissues.

*Sarcodia montagneana* is a species of red algae that belongs to the family *Sarcodiaceae*, also known as *S. ceylanica* or *S. suiae*. Previous studies have shown the anti-inflammatory, antioxidative, and antitumor effects of the extract of *S. montagneana* (SME) [6,7,8,9]. The growing season of *S. montagneana* takes place throughout the entire year in the seaside areas of East Asia. Moreover, artificial cultivation skills have also been successfully developed [6,10]. These reasons make *S. montagneana* a potential food-based supplement for health management. Many dietary-derived composites are demonstrated to have significant immune modulation functions. Our previous study has also shown the potent anti-inflammatory and pain-relieving properties of freshwater clams (*Corbicula fluminea*) [11]. Freshwater clam extract (FCE) has been widely investigated as an effective immune regulator for attenuating inflammatory responses. Its hepatoprotective function and suppression of the release of pro-inflammatory cytokines, such as TNF-α and IL-1β, were also proven in several studies [12,13,14]. 

The aim of this research is to explore the potential synergistic benefits of combining SME and FCE in managing osteoarthritis symptoms. Specifically, this study seeks to investigate the anti-inflammatory properties of these compounds through in vitro testing and the MIA-induced OA model. The data shown here could provide crucial insights into using the combination of SME and FCE as a therapeutic solution for alleviating OA symptoms.

## 2. Materials and Methods

### 2.1. Cell Culture and Treatment

Cells from the pro-monocytic cell line THP-1 were obtained from the Food Industry Research and Development Institute (BCRC, Hsinchu, Taiwan) and cultured in RPMI 1640 (Thermo Fisher Scientific, Waltham, MA, USA) supplemented with 10% fetal bovine serum (Hyclone), 0.05 mM β-mercaptoethanol (Sigma-Aldrich, St. Louis, MO, USA), 2 g/L sodium bicarbonate, and 1% penicillin/streptomycin (PS, Biological Industries, Beithemeek, Israel). The cells were maintained in 5% CO_2_ at 37 °C. The cells were divided into the following treatment groups: cells without LPS (Sigma) as the control group; cells treated with LPS (1 μg/mL) alone; and cells treated with different concentrations of the extracts of *S. montagneana* and *Corbicula fluminea* in the presence of LPS. The supernatants from the cells treated for 24 h were harvested and analyzed via an enzyme-linked immunosorbent assay (ELISA).

### 2.2. Preparation of the Extracts of Sarcodia montagneana and Corbicula fluminea

Sarcodia montagneana (provided by Professor Chang-Jer Wu of National Taiwan Ocean University, Keelung, Taiwan) was first processed into a fine-grained powder with a particle size of 80 mesh (each particle size was approximately 180 μm) using a homogenizer. The SM powder was added to 15 times its volume of H_2_O, boiled to 100 °C for 10 min, and maintained at 75 °C for 6 h of direct drying or 10–20 min of reduced pressure concentration. Then, the SM powder was obtained for further experiments. Following this, the components of the SM powder were determined via high-performance liquid chromatography–gel permeation chromatography (HPLC-GPC). The HPLC system consisted of a PU-1580 pump unit from Jasco (Tokyo, Japan) and a gel permeation chromatography (GPC) column (OHpak SB-804, 806 HQ) (Shodex, Showa Denko, Tokyo, Japan). Sarcodia montagneana water extract (SMw) (1 mg) was dissolved in 1 mL of deionized water. The mobile phase for HPLC was deionized water with a flow rate of 0.8 mL/min. The retention time was recorded using EC 2000 GPC software. Calibration curves were prepared using standards purchased from Sigma-Aldrich. The significant components of the polysaccharides derived from the SM powder are shown in Supplementary Figure S1. The ratio of whole freshwater clam powder (ZH-Biotech, Taipei, Taiwan) to chloroform was 1:10 (*w*/*w*). After 12 h of rest at room temperature, the mix was filtered through No. 1 filter paper (Toyo Roshi Kaisha, Tokyo, Japan) and centrifuged at 4500× *g* for 20 min to eliminate insoluble material. The resulting supernatant was filtered through No. 1 filter paper a second time, followed by drying in a rotation evaporator under a vacuum. The final product was dissolved in dimethyl sulfoxide (DMSO) for analysis.

### 2.3. LPS-Induced Systemic Inflammation in C57BL/6JNarl Mice

To evaluate inflammation, cartilage regeneration, and synovial repair in an animal model, 4- to 6-week-old C57BL/6JNarl mice were acquired from the National Laboratory Animal Center (Taipei, Taiwan). The mice were housed in the Laboratory Animal Center of the National Yang-Ming Chiao Tung University. They had access to food and water ad libitum under a 12/12 h light/dark cycle. The experimental protocol was approved by the Institutional Animal Care and Use Committee, National Yang-Ming Chiao Tung University, Hsinchu, Taiwan (IACUC No. NCTU-IACUC-108004). 

### 2.4. LPS-Induced Inflammatory Model

#### 2.4.1. Preventive Model

**Figure 1:**
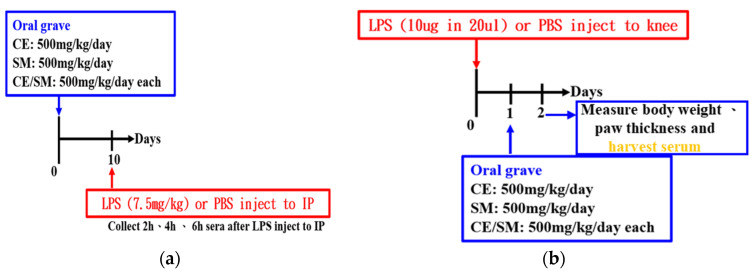
The protocol for investigating the synergistic effects of SME and FCE on (**a**) preventing systemic inflammation and (**b**) alleviating mouse paw swelling induced by LPS.

Before the LPS induction of systemic inflammation, the mice were randomly divided into four groups (n = 3 for each group): LPS (LPS injection), LPS + FCE (LPS injection following 500 mg/kg/day O.P. treatment), LPS + SME (500 mg/kg/day), and LPS + SME + FCE. Then, an intraperitoneal injection of LPS (7.5 mg/kg) was administered to induce a systemic inflammatory response. Blood samples were collected from the mice at post-injection times of 2, 4, and 6 h. Then, the amount of TNF-α in serum was quantified using an R&D human TNF-α ELISA kit (Figure 1a).

#### 2.4.2. Treatment Model

Injection of LPS (10 μg/20 μL) into the knee joints of the mice was performed on Day 0. After 24 h of rest, the mice were randomly divided into four groups (n = 3 for each group): LPS (LPS injection), LPS + FCE (LPS injection following 500 mg/kg/day O.P. treatment), LPS + SME (500 mg/kg/day), and LPS + SME + FCE. Continuous oral gavage of 4 different groups was routinely monitored. Meanwhile, the body weights and paw thicknesses of the mice were recorded daily (Figure 1b).

### 2.5. MIA-Induced Mouse OA Model

#### 2.5.1. Preventive Model

**Figure 2:**
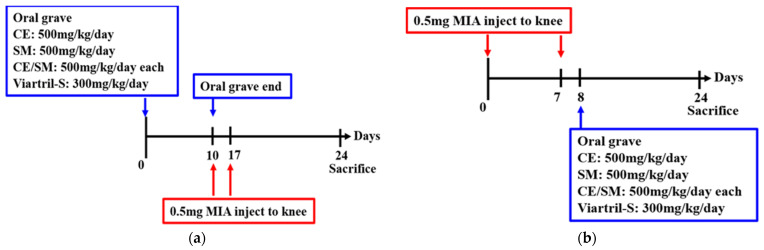
The protocol for investigating the synergistic effects of SME and FCE on (**a**) preventing MIA-induced OA in mice and (**b**) alleviating the symptoms of MIA-induced OA.

To assess the preventive efficacy of SME and FCE, consecutive 10-day feeding before MIA-induced osteoarthritis was established. Oral gavage of the following 4 formulations was administered simultaneously every day from Day 0 to Day 10: (i) FCE 500 mg/kg/day; (ii) SME 500 mg/kg/day; (iii) FCE/SME 500 mg/kg/day; and (iv) Viartril-S (Rottapharm, Monza, Italy) 300 mg/kg/day. After the preventive treatment, 0.5 mg of MIA was injected into the knee joints of the mice to induce acute osteoarthritis. Then, 7 days later (on Day 17), a booster MIA injection was administered to ensure uniform OA symptoms (Figure 2a).

#### 2.5.2. Treatment Model

The first 0.5 mg MIA injection and the booster with the same dose were administered on Day 0 and Day 7, respectively. From Day 8 to Day 24, oral gavage of 4 different groups of formulations were administered: (i) FCE 500 mg/kg/day; (ii) SME 500 mg/kg/day; (iii) FCE/SME 500 mg/kg/day; and (iv) Viartril-S (Rottapharm) 300 mg/kg/day. For inflammation, the condition of paw edema was recorded every 3 days. Proper euthanasia was conducted on Day 24, and then, cartilage and bone tissue were examined via histopathological assessment with H&E and Safranin O staining (Figure 2b).

### 2.6. Histopathology of Cartilage Regeneration and Inflammation

The knee joints were fixed in 10% neutral formalin for seven days, and then, decalcified in rapid decalcifying solution (Sigma) for five days before being harvested and embedded in paraffin wax. The deparaffinized slides were then sagittally sectioned at 4 μm and stained with Hematoxylin and Eosin (H&E) and Safranin O/Fast green stain. 

### 2.7. ELISA

The supernatant from the treated THP-1 cells and sera collected from the experimental mice on Day 21 were collected for measuring IFN-*γ* (Cat. No. DY285B-05, R&D system, Minneapolis, MN, USA) and TNF-α. Human and mouse TNF-α concentrations in the supernatant or serum were evaluated using ELISA kits (Cat. No. DY 210-05 (Human), DY 210-05 (mice), R&D system), following the manufacturer’s protocol.

### 2.8. Statistical Analysis

Data are expressed as the mean ± standard deviation (SD) in this study. GraphPad Prism 9.0 software was utilized for graphing and analyzing the ELISA results, and changes in body weight were analyzed via two-way ANOVA, followed by Tukey’s post hoc test. The scoring analysis was conducted using the Kruskal–Wallis test and Dunn’s post hoc test. A *p*-value of less than 0.05 was considered statistically significant.

## 3. Results

### 3.1. SME and FCE Reduced IFN-γ and TNF-α Production Induced by LPS-Treated THP-1 Cells

FCE was prepared via chloroform purification as described in our previous study [14]. For SME preparation, after the homogenous processing of *S. montagneana* for 6 h, the total sugar and sulfate contents of SME were examined and recorded to two decimal places. The total sugar content of SME was 324.55 ± 31.01 mg/g, while the sulfate group content was 23.70 ± 1.26%. The values shown above are the means ± SDs from triplicate independent experiments. 

**Figure 3:**
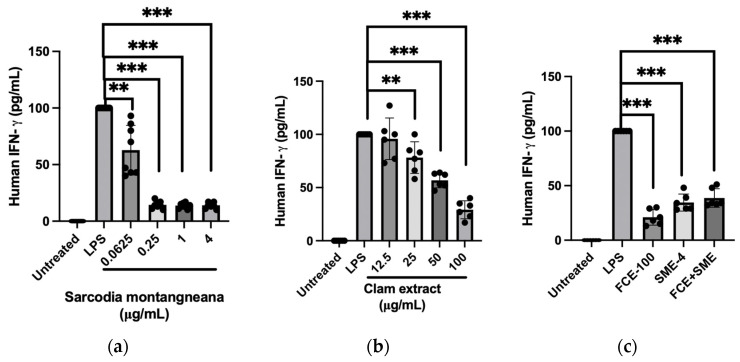
The synergistic effects of combining the extracts of *S. montagneana* and freshwater clams significantly inhibit LPS-induced IFN-*γ* secretion from THP-1 cells. Treatments were as follows: (**a**) extract of *S. montagneana* (SME); (**b**) extract of *Corbicula fluminea *(FCE); and (**c**) SME + FCE. The data are expressed as the mean ± SD of three determinations. (**) *p* < 0.01 and (***) *p* < 0.001 versus LPS alone.

LPS (1 μg/mL)-treated THP-1 cells induced a significant amount of IFN-*γ* (98.3 ± 0.2 pg/mL). The serial dosage of the SME treatment significantly suppressed the IFN-*γ* concentration, even at the lowest dosage of 0.0625 mg/mL SME (59.6 ± 9.5 pg/mL, *p* < 0.01) (Figure 3a). In treating the LPS-incubated THP-1 cells, FCE decreased the induction of IFN-*γ* by LPS in a dose-dependent manner (12.5, 25, 50, and 100 μg/mL) (Figure 3b). 

The above results show that both SME and FCE can inhibit the LPS-induced production of IFN-*γ*. We further validated whether the combination of these two extracts could have synergistic effects. The following experiment combined the highest concentrations of both the FCE and SME groups. Compared to the positive control group (LPS administration), both single treatments of 100 μg/mL FCE and 4 mg/mL SME showed a lower level of IFN-*γ*, as did the combination of FCE and SME (Figure 3c).

**Figure 4:**
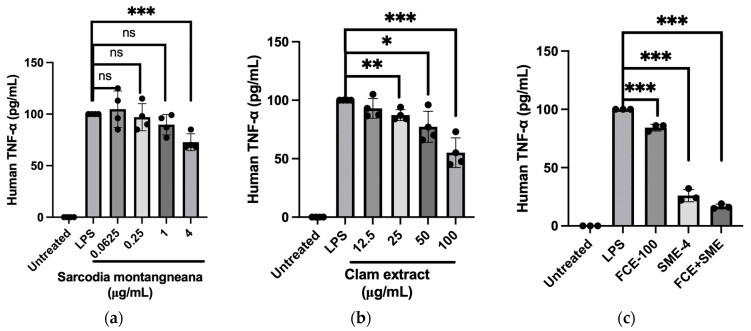
The synergistic effects of combining the extracts of *S. montagneana* and freshwater clams significantly inhibit LPS-induced TNF-α secretion from THP-1 cells. Treatments were as follows: (**a**) extract of *S. montagneana* (SME); (**b**) extract of *Corbicula fluminea* (FCE); and (**c**) SME + FCE. The data are expressed as the mean ± SD of three determinations. ns: non-significant; (*) *p* < 0.05, (**) *p* < 0.01, and (***) *p* < 0.001 versus LPS alone.

Similar effects were also observed in the amount of TNF-α induced by the LPS-treated THP-1 cells. LPS-treated THP-1 cells induced a significant amount of TNF-α (99.2 ± 0.3 pg/mL). The serial dosage of the SME treatment gradually suppressed the TNF-α concentration, especially at 4 mg/mL SME (69.3 ± 8.4 pg/mL, *p* < 0.001) (Figure 4a). In treating the LPS-incubated THP-1 cells, FCE decreased the induction of TNF-α by LPS in a dose-dependent manner (12.5, 25, 50, and 100 μg/mL) (Figure 4b). In the co-treatment with SME and FCE, compared to the LPS administration group, though both single treatments of 100 μg/mL FCE and 4 mg/mL SME showed a lower level of TNF-α, the synergic effect of the combination of FCE and SME significantly reduced the amount of TNF-α (Figure 4c).

### 3.2. The Extracts of S. montagneana and Corbicula fluminea Protected the Mice from Systemic Inflammation and Reduced Paw Swelling Induced by LPS

**Figure 5:**
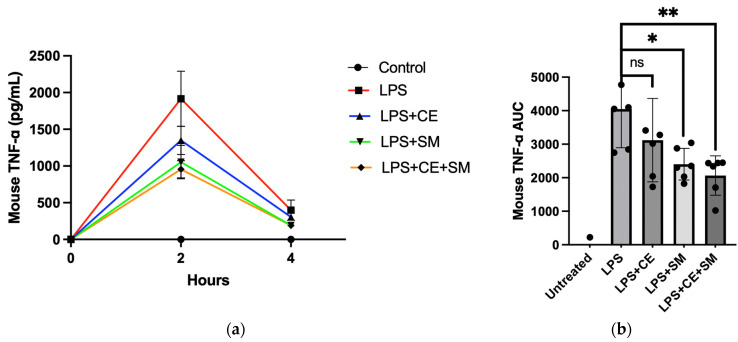
The extracts of *S. montagneana* and *Corbicula fluminea* protected mice from LPS-induced systemic inflammation. After 10-day feeding of SME and FCE, the mice that received an intraperitoneal injection of LPS showed a significantly lower TNF-α concentration in their plasma. (**a**) Plasma TNF-α levels at 2, 4, and 6 h after LPS treatment; (**b**) AUC of the TNF-α concentration. The data are expressed as the mean ± SD of three determinations. ns: non-significant; (*) *p* < 0.05 and (**) *p* < 0.01 versus LPS alone.

The protective efficacy of SME and FCE was first investigated to determine whether they could prevent LPS-induced systemic inflammation in the mice; this was determined by measuring the TNF-α concentration in mouse sera. The area under the curve was also calculated in this experiment. While the results of FCE and SME showed a mild decrease in TNF-α 2 and 4 h post-injection of LPS, there was no statistical significance in each group (Figure 5a). Only when the mice were administered both FCE and SME (500 mg/kg) for 10 days did their plasma TNF-α concentrations significantly decrease, even in the presence of in vivo LPS stimulation (Figure 5a,b).

**Figure 6:**
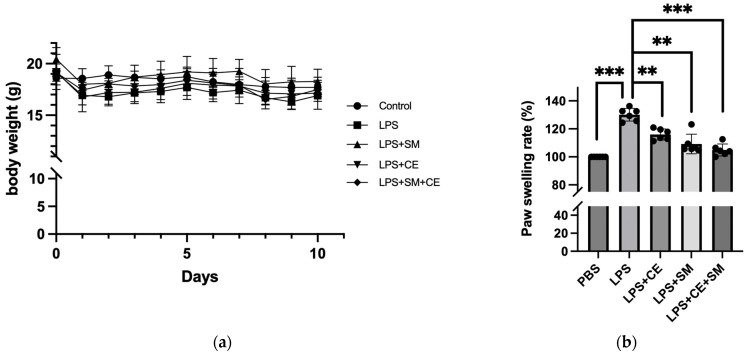

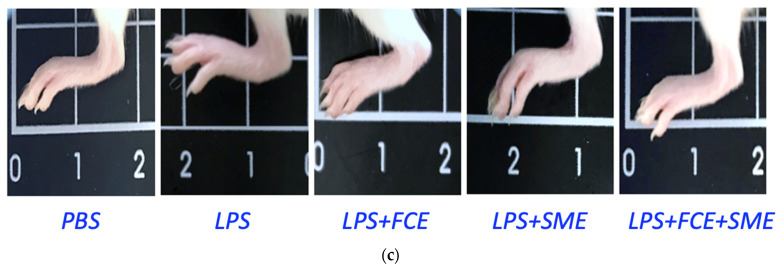
The extracts of *S. montagneana* and *Corbicula fluminea* reduced LPS-induced paw swelling. (**a**) The combination of SME and FCE reversed LPS-induced body weight loss compared to the LPS-treated group (*p* < 0.05). (**b**) FCE, SME, and FCE + SME reduced the thickness of paw swelling, and the combinatory treatment was the most effective. (**c**) Representative photos of each treatment. The data are expressed as the mean ± SD of three determinations. (**) *p* < 0.01 and (***) *p* < 0.001 versus LPS alone.

We then explored whether the combined usage of SME and FCE could also relieve the pre-existing inflammatory condition in the mice. After Day 0 of LPS injection, all groups’ body weight loss rates were higher than 10%, except the group with the FCE/SME combination. The FCE/SME combination group had only 5.66% body weight loss, which was statistically different from the control (LPS injection) group (Figure 6a). Meanwhile, the single FCE and SME treatments and their combination were administered to mitigate the clinical signs of mouse paw swelling. Among all the treatments, the LPS with FCE treatment and LPS with FCE/SME treatment presented significant anti-inflammation effects. The results showed 3.42% and 1.55% paw swelling, respectively. The latter treatment group (LPS with CE/SM) also demonstrated the best alleviation of symptoms of clinical swelling (Figure 6b,c).

### 3.3. Preventive and Relieving Effects of SME and FCE in MIA-Induced Osteoarthritis Model

**Figure 7:**
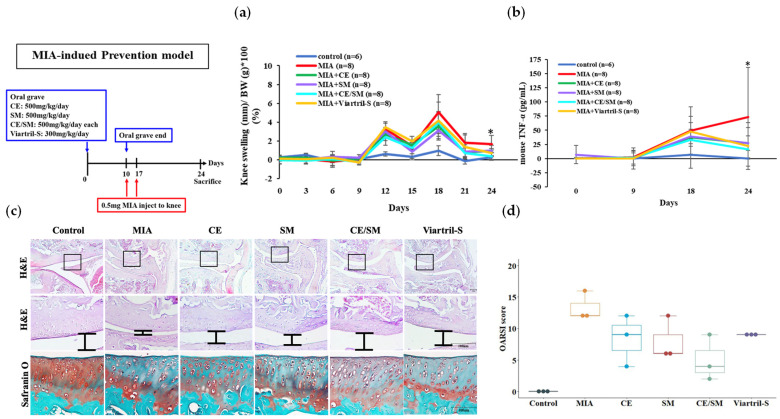
The extracts of *S. montagneana* and *Corbicula fluminea* protected mice from MIA-induced osteoarthritis. On Day 24, the mice were sacrificed for the following analysis. The combination of SME and FCE significantly reduced (**a**) knee swelling and (**b**) plasma TNF-α in MIA-treated knee osteoarthritis. (**c**) SME + FCE significantly protected the cartilage from proteoglycan loss and (**d**) led to the lowest OSARI score. The data are expressed as the mean ± SD of three determinations. (*) *p* < 0.05.

The mice were randomly divided into four groups receiving the following formulations from Day 0 to Day 10 before MIA administration: (i) FCE 500 mg/kg/day; (ii) SME 500 mg/kg/day; (iii) FCE/SME 500 mg/kg/day; and (iv) Viartril-S (Rottapharm, Monza, Italy) 300 mg/kg/day. The apparent rising curve between Day 9 and Day 12 of knee swelling demonstrated the consistency and success of the MIA-induced OA model. The peak of inflammation appeared on Day 18, followed by a recession in all the groups. The following observations indicate that the anti-inflammation effect of FCE/SME was present from Day 18 to Day 24, but it only showed significant differences on the Day 24 measurement. Moreover, the knee swelling percentage of the FCE/SME group dropped significantly on Day 21 and Day 24 (Figure 7a). The mice fed FCE + SME were also found to have decreased TNF-α in the plasma, with a similar level to those fed Viartril-S (Figure 7b).

Safranin O staining indicates the proteoglycan content, which is abundant in healthy cartilage. The intensity of pink staining is proportional to the amount of proteoglycan present. As MIA-induced OA progresses, there is a reduction in the proteoglycan content, resulting in a decreased intensity of Safranin O staining. As shown in Figure 7c, the proteoglycan loss was partially restored when the OA mice were treated with FCE, SME, or Viatril-S alone. However, the most significant effect was observed in the SME + FCE group. 

Further, to ensure a standardized and reliable assessment of histopathological OA, we utilized the OARSI scoring system to comprehensively evaluate cartilage damage and regeneration in the knee joint. A semi-quantitative scoring method was adopted, where six grades (ranging from 0 to 6) and four stages (ranging from 0 to 4) were applied to assess the depth progression of the cartilage and the extent of joint involvement. The OA score was calculated as a product of the grade and stage, with a range of 0–24. Our results showed that feeding mice SME and FCE led to the lowest OSARI score, which means that the combination of these two extracts could effectively prevent MIA-induced osteoarthritis in the joints (Figure 7d). 

**Figure 8:**
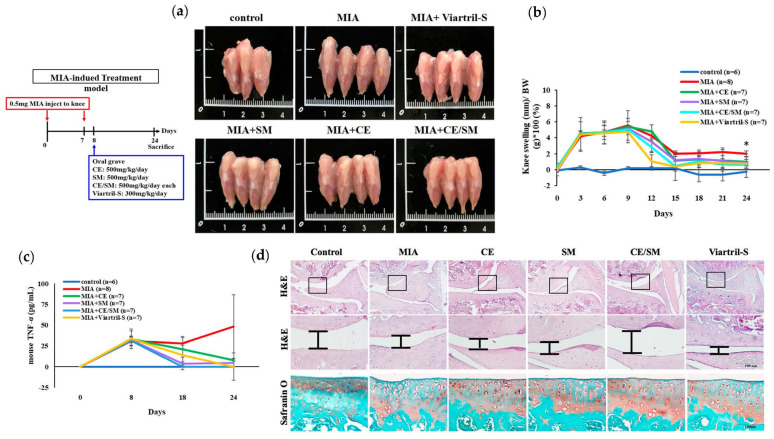
The extracts of *S. montagneana* and *Corbicula fluminea* alleviated MIA-induced osteoarthritis. On Day 24, the mice were sacrificed for the following analysis. The combination of SME and FCE significantly reduced (**a**,**b**) knee swelling and (**c**) plasma TNF-α in MIA-treated knee osteoarthritis. (**c**) SME + FCE significantly protected the cartilage from proteoglycan loss and (**d**) led to the lowest OSARI score. The data are expressed as the mean ± SD of three determinations. (*) *p* < 0.05.

In mice suffering from MIA-induced OA (Figure 2b), similar results were found, showing that, even when OA was progressive, the combined treatment of SME and FCE significantly inhibited the TNF-α level, repaired the cartilage, and restored the function of the damaged knee joints (Figure 8).

## 4. Discussion

Inflammation plays a significant role in the progression of osteoarthritis. The inflammatory response in OA has several detrimental effects on joint tissues and exacerbates the disease process, such as the breakdown of articular cartilage. Pro-inflammatory cytokines, including interleukin (IL)-1β and TNF-α, are released by activated macrophages [15,16] to promote the production of enzymes, including matrix metalloproteinases (MMPs), which degrade the extracellular matrix of cartilage and weaken the cartilage structure, leading to its erosion and loss over time [17]. Further, inflammatory responses induce changes in the subchondral bone. Inflammatory cytokines stimulate the production of osteoclasts, which are cells responsible for bone resorption. This excessive bone resorption alters the balance between bone breakdown and formation, leading to a net loss of bone density and structural integrity. Moreover, the release of inflammatory factors by activated osteoclasts further perpetuates the inflammatory response in the joint [18].

Additionally, inflammation in OA triggers the release of pain-inducing substances, such as prostaglandins and bradykinin. These substances sensitize nerve endings within and around the joint, increasing pain perception. Inflammatory cytokines also activate pain receptors in the joint tissues, contributing to pain and discomfort experienced by individuals with OA [19]. When chronic inflammation happens in OA patients, it can lead to the development of synovitis, characterized by inflammation of the synovial membrane lining the joint. The inflamed synovium produces excessive synovial fluid, causing joint swelling and further exacerbating joint stiffness and pain [20]. Taken together, the pathogenesis of OA is complicated, and at present, there are few therapeutic choices its effective treatment. Preventing or interrupting the vicious cycle between inflammation and joint tissue damage should be the solution to alleviating the progression of OA.

Marine natural products have seen significant growth in development, with algal products being a significant component of this field due to being regenerative, edible, and environmentally friendly. Red algae, in particular, have higher biodiversity than green and brown algae, along with the potential to be used for developing new drugs. Previous studies showed that *S. montagneana* is able to reduce triglyceride levels in rats, and its water extract can eliminate free radicals and possess anti-diabetic properties [21]. Further an investigation also disclosed that the ethyl acetate extract of *S. montagneana* decreased LPS-induced iNOS protein expression in RAW 264.7 cells and suppressed pro-inflammatory mediators, such as iNOS, IL-1β, and myeloperoxidase (MPO), in carrageenan-induced rat paw edema [6]. The present data demonstrate that the water extract of *S. montagneana*, with polysaccharides and sulfate contents as the major components, can be used to effectively suppress IFN-*γ* and TNF-α secretion in vitro and in vivo, relieve paw swelling, and prevent or reverse cartilage damage in MIA-induced OA mice. 

*Corbicula fluminea* extract has been shown to possess a range of potential beneficial effects on human health due to its diverse bioactive compounds. Studies have indicated that *C. fluminea* extract may have anti-inflammatory, antioxidant, muscle-building, and hepatoprotective effects. Our previous study showed that the combination of FCE and curcumin could reverse OA in rats [11]. Further, when pre-diabetic patients received FCE 2 g/day for 180 days, the serum TNF-α level was significantly reduced [22]. *Corbicula fluminea* extract has also been found to possess significant antioxidant activity. The data reveal that *C. fluminea* extract can protect liver cells against oxidative damage induced by alcohol treatment [23]. Furthermore, it is also reported that FCE has protective effects against oxidative stress in cells exposed to hypoxia/reoxygenation injury [24]. For OA treatment, inhibiting inflammation is the key to preventing disease progression. However, having enough muscle fibers surrounding and stabilizing the joint is also necessary. *C. fluminea* extract could promote muscle protein synthesis and the expression of muscle growth factors in rats [25]. 

## 5. Conclusions

The combined application of *S. montagneana* and *C. fluminea* extracts for the maintenance or improvement of human health was carried out and analyzed in this study. The high yield, affordable price, nutritious compositions, and antioxidative and anti-inflammatory effects of both extracts provide a new solution for developing OA therapies. 

## Data Availability

Data supporting this study are included within the article and Supplementary Materials.

## Supplementary Materials

The following supporting information can be downloaded at: https://www.mdpi.com/article/10.3390/antiox12122068/s1, Figure S1. GPC spectrum of *Sarcodia montagneana* water extract (SMw). Column: Ohpak SB-804HQ and Ohpak SB-806HQ, size exclusion column: 8.0 mmID × 300 mm; column temperature: 35 °C; injection volume: 20 μL: flow rate: 0.8 mL/min; mobile phase: H_2_O; detector: RI (Refractive index); Figure S2. Antioxidant activity of Sarcodia montagneana extracts. (a) Superoxide anion radical scavenging activity of gallic acid at different concentrations as the standard, and (b) superoxide anion radical scavenging activity of SMw and SMe at different concentrations. (c) DPPH (α,α-diphenyl-β-pricrylhydrazyl) free radical scavenging activity of ascorbic acid at different concentrations as the standard, and (d) DPPH free radical scavenging activity of SMw and SMe at different concentrations. (e) Ferrous ion chelating activity of EDTA at different concentrations as the standard, and (f) ferrous ion chelating activity of SMw and SMe at different concentrations. The results are expressed as the mean ± SD. Reference [26] is cited in the Supplementary Materials.
