# Supplementary material for: The Synergistic Effects of Corbicula fluminea and Sarcodia montagneana on Alleviating Systemic Inflammation and Osteoarthritis Progression
Source: Antioxidants (Basel). 2023 Dec 1;12(12):2068. doi: 10.3390/antiox12122068 (PMC10740935; doi:10.3390/antiox12122068)
Supplement: Supplementary file 1 [file antioxidants-12-02068-s001.zip › antioxidants-2645189-supplementary.pdf]

Supplementary Data

Supplementary methods

1. *DPPH radical scavenging assay*

The radical scavenging ability of *Sarcodia montagneana* extract was assessed using the method of Shimada et al. with slight modifications [1]. The radical scavenging ability of the extract was determined at a concentration of 10 mg/mL in ethanolic DPPH solution (0.1 mM) (Sigma, Saint Louis, USA). In the control, water was used in place of the sample. Cuvettes were left in the dark at room temperature for 30 min, and the resulting color was measured spectrophotometrically at 517 nm against blanks. A decreasing intensity of purple color was considered to be related to greater radical scavenging ability, which was calculated using the following equation: DPPH radical scavenging ability= $[1- (A_{S:30}/A_{B:30})] \times 100$, where $A_{S:30}$ is the absorbance of the sample and $A_{B:30}$ is the absorbance of the blank at 30 min reaction time.

2. *Superoxide anion scavenging activity*

The NADH/PMS/NBT system was used to determine the superoxide anion scavenging activity of *Sarcodia montagneana* extract. Superoxide radicals were generated in the PMS–NADH system through the oxidation of NADH and assayed by reducing nitroblue tetrazolium (NBT) (Sigma). Briefly, 50 μL of NBT solution (300 μM in 100 mM phosphate buffer, pH 7.4), 50 μL of NADH solution (936 μM in 100 mM phosphate buffer, pH 7.4), and 50 μL of sample solution (2.5 mg/ml in distilled water) were mixed. The reaction was started by adding 50 μL of phenazine methosulphate (PMS) solution (120 μM in 100 mM phosphate buffer, pH 7.4) to the mixture. The reaction mixture was incubated at room temperature for 5 min, and the absorbance at 560 nm was measured against blank samples. A decrease in the absorbance of the reaction mixture indicated increased superoxide anion scavenging activity. NADH/PMS/NBT solution without the sample solution was used as the control. The percent inhibition of superoxide anion generation was calculated using the following formula: Scavenging activity (%) = $[1- (A_{S:5}/A_{B:5})] \times 100$, where A_S is the absorbance of the sample and A_B is the absorbance of the blank at 5 min reaction time.

Supplementary results

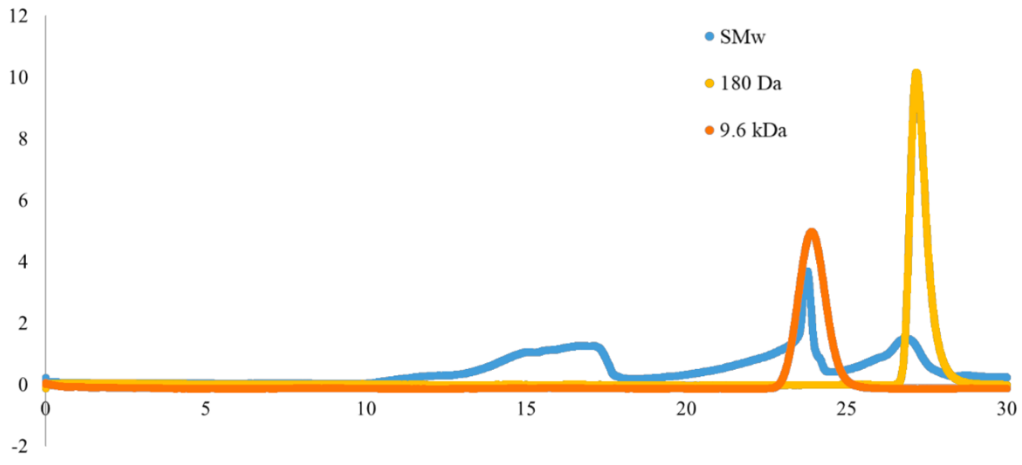

Supplementary Figure S1. GPC spectrum of *Sarcodia montagneana* water extract (SMw). Column: Ohpak SB-804HQ and Ohpak SB-806HQ, size exclusion column: 8.0 mmID × 300 mm; column temperature: 35°C; injection volume: 20 μL: flow rate: 0.8 mL/min; mobile phase: H2O; detector: RI (Refractive index).

The extract of S. montagneana possesses effective antioxidant activity

The extract of *Sarcodia montagneana* was subjected to antioxidant capacity testing, including measurements of superoxide anion radical scavenging ability, DPPH radical scavenging ability, and ferrous ion chelation capacity. Supple. Figure 2a,b show the superoxide anion radical scavenging ability, with the former showing gallic acid as the standard and the latter showing the ability of SME (SMw: water extract; SMe: ethanol extract) to eliminate superoxide anion radicals. At an SMw concentration of 250 μg/mL, the antioxidant capacity was roughly equivalent to that of the standard at 500 μg/mL, while at 1000 μg/mL, it matched the standard's antioxidant capacity at 62.5 μg/mL. Supple. Figure 2c,d illustrate the DPPH radical scavenging ability, with the former showing the standard, ascorbic acid, and the latter showing the ability of *S. montagneana* to eliminate DPPH radicals. At an SMw concentration of 62.5 μg/mL, the scavenging ability was approximately equivalent to that of the standard at 15.625 μg/mL. Supple. Figure 2e,f demonstrates the ferrous ion chelation capacity, with the former showing the standard, EDTA, and the latter indicating the sample's ability to chelate ferrous ions. At an SMw concentration of 250 μg/mL, the chelation capacity was roughly equivalent to that of the standard at 50 μM, while at SMe 500 μg/mL, it matched the standard's chelation capacity at 25 μM. These results for the three types of antioxidant capacity indicate that the higher the SME concentration, the better the antioxidant ability, displaying a dose-dependent relationship.

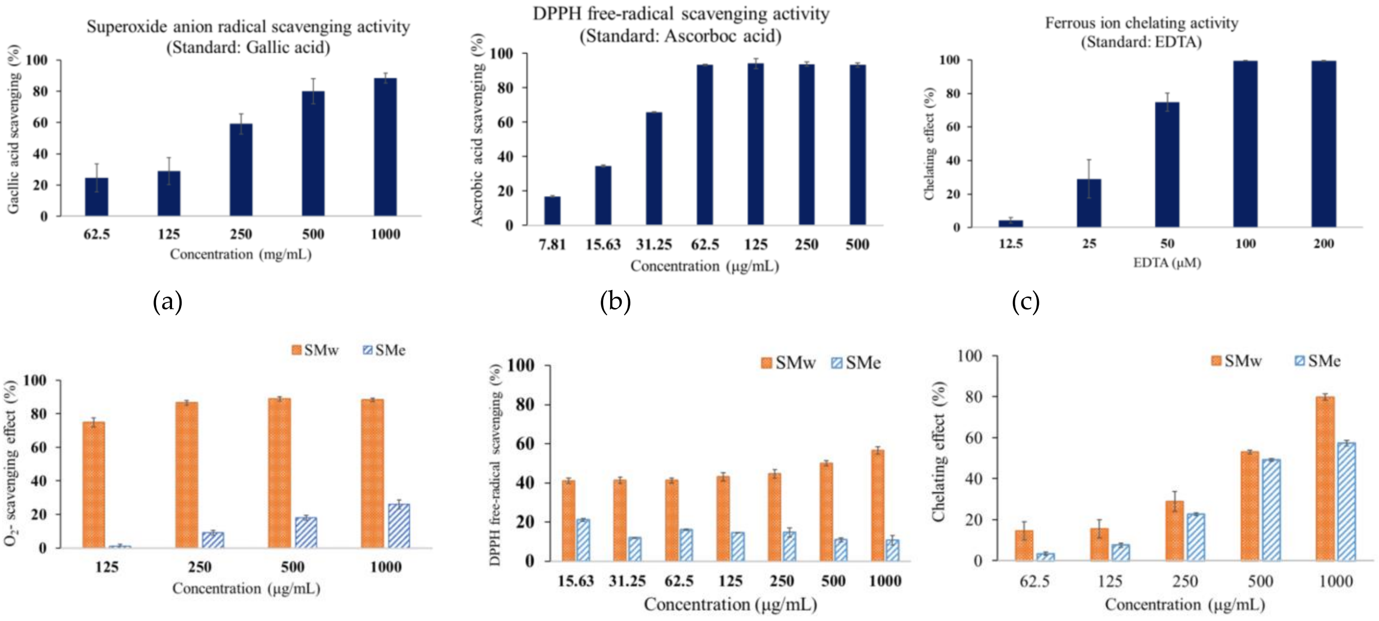

(a) (b) (c)

(d) (e) (f)

Supplementary Figure S2. Antioxidant activity of *Sarcodia montagneana* extracts. (a) Superoxide anion radical scavenging activity of gallic acid at different concentrations as the standard, and (b) superoxide anion radical scavenging activity of SMw and SMe at different concentrations. (c) DPPH (α,α-diphenyl-β-pricrylhydrazyl) free radical scavenging activity of ascorbic acid at different concentrations as the standard, and (d) DPPH free radical scavenging activity of SMw and SMe at different concentrations. (e)

Ferrous ion chelating activity of EDTA at different concentrations as the standard, and (f) ferrous ion chelating activity of SMw and SMe at different concentrations. The results are expressed as the mean ± SD.

References

1. Shimada K, Fujikawa K, Yahara K, Nakamura T: **Antioxidative Properties of Xanthan on the Autoxidation of Soybean Oil in Cyclodextrin Emulsion**. *J Agr Food Chem* 1992, **40**(6):945-948.
